# Sexual practices and HPV infection in unvaccinated young adults

**DOI:** 10.1038/s41598-022-15088-8

**Published:** 2022-07-20

**Authors:** Sílvia Pauli, Natália Luiza Kops, Marina Bessel, Luisa Lina Villa, Flávia Moreno Alves Souza, Gerson Fernando Mendes Pereira, Fernando Neves Hugo, POP-Brazil Study Group, Juliana Comerlato, Isabel Bandeira, Bruna Fernandes, Tiago Fetzner, Milena Mantelli Dall Soto, Thais Baptista, Barbara Pereira Mello, Giana Mota, Eliana Wendland

**Affiliations:** 1 Hospital Moinhos de Vento, Rua Ramiro Barcelos 910, Porto Alegre, RS 90035-004 Brazil; 2 Department of Radiology and Oncology, Medical School, Universidade de São Paulo (USP), and Instituto do Câncer do Estado de São Paulo (ICESP), São Paulo, SP Brazil; 3 Department of Chronic Conditions and Sexually Transmitted Infections, Ministry of Health, Brasília, DF Brazil; 4 Department of Preventive and Social Dentistry, Universidade Federal do Rio Grande do Sul, Porto Alegre, RS Brazil; 5 Department of Public Health, Universidade Federal de Ciências da Saúde de Porto Alegre (UFCSPA), Porto Alegre, RS Brazil

**Keywords:** Cancer prevention, Epidemiology

## Abstract

The present study aimed to evaluate the association of genital and oral HPV infection among different sexual practices in both sexes. In total, 6388 unvaccinated men and women aged 16–25 years from all state capitals of Brazil were enrolled in through primary care services between September 2016 and November 2017. Genital and oral HPV genotyping was performed using the Roche Linear Array. Poisson regression analysis with robust variance was conducted to examine factors associated with overall HPV infection. A higher prevalence of genital HPV was found in women who practiced oral sex (57.85%) and in men who practiced all types of sex (65.87%). However, having more sexual partners and being younger were more important than the type of sex practiced. HPV 6 (7.1%) and 16 (10.5%) were significantly more prevalent in women who practiced oral sex, while HPV 6 (23.96%) and 11 (21.49%) were more prevalent in men who practiced anal sex. The type of sex was not associated with oral HPV prevalence. Genital and oral HPV infection were not associated by different sexual practices in the studied population.

## Introduction

Human Papillomavirus (HPV) infection is considered a risk factor for cancer in different sites of the body, such as the cervix, penis, vulva, and oropharynx^1^. The probability of acquiring HPV during one’s life has been estimated to be 84.6% for women and 91.3% for men^2^. While 90% of these infections are cleared within two years, mainly as a result of cell-mediated immune responses directed against early HPV proteins^3^, some types of the genus Alphapapillomavirus (alpha-HPV) can cause anogenital cancers^4^.

These alpha-HPV infect mainly the basal epithelial cells of the anogenital mucosa via microabrasions in the epithelial lining^5,6^. For this reason, horizontal transmission occurs mainly through the sexual act—sexual penetration or intimate genital contact^7^—although it may also occur through kissing^8^ or hand-to-genital contact ^9^. The probability of HPV transmission per sex act seems to be around 40% (range 5–100%)^10^. Multiple HPV genotype infections are common, and genotype-specific concordance of HPV 6, 11, 16, and 18 was 25.5% in a meta-analysis focusing on 30 studies and 2972 couples^11^.

Transmission of HPV to the oral cavity and the consequent risk of oropharyngeal cancer is increased in women with cervical infection and in their partners, suggesting possible transmission between the oral cavity and the genitals^12^. In addition, a higher transmission rate of genital HPV seems to occur from women-to-men than men-to-women contacts, as shown by a systematic review and meta-analysis that synthesized data of 752 heterosexual couples^13^. However, many gaps still exist in our understanding of HPV transmission between different sites of the body, such as whether mouth-genitalia transmission is different from genitalia-genitalia transmission or whether different virus types have preferences for specific sites^14^.

There are also other possible non-sexual transmission routes. Men who have sex with women with prior genital HPV infections also had a higher risk of a subsequent type-specific anal infection independent of sexual intercourse with female partners^15^. The authors concluded that autoinoculation is a possible mechanism^15^. Both the presence of the virus in these two anatomic sites and the lack of viral concordance in some people could be due to genetic predisposition, conditions of altered immune response, host defense, or local cellular factors^15^.

The determinants of exposure to HPV seem to be similar to those for most sexually transmitted infections^7^. However, understanding the transmission dynamics of HPV and identifying different behaviors associated with an increased risk of HPV infection in the young population is of pivotal importance to promote adequate planning of public policies. Such findings may help health care providers to better counsel patients on safe sex practices and promote sexual health.

Although the overall prevalence of both genital and oral sites are well described in the Brazilian population^16–19^, few studies have assessed the role of different sexual practices in HPV infection, especially in both men and women from the same underlying population^20^. Therefore, the present study aimed to evaluate the association of genital and oral HPV infection based on different sexual practices in both sexes.

## Methods

### Study design and population

We analyzed data from unvaccinated participants in the POP-Brazil study, a cross-sectional study with sexually active women and men aged 16–25 years from 26 Brazilian state capitals and the Federal District. Exclusion criteria were pregnancy, history of hysterectomy or trachelectomy, and history of cervical intraepithelial neoplasia grade 2 or higher.

The participants were recruited in primary care services using different approaches such as personal invitations during routine healthcare visits and home visits, as well as outreach using patient lists and local media, between September 2016 and November 2017.

This study was approved by the Ethics Committee of Hospital Moinhos de Vento (no. 1607032) and the committees from the collaborator’s centers. All study participants provided written consent after being informed about the study procedures.

### Study variables

All participants answered face-to-face interviews with questions about sociodemographic factors, alcohol and drug use, sexual behaviors, and lifetime history of STIs.

The demographic and socioeconomic variables investigated were sex (female or male), age (< 22 and ≥ 22 years), self-reported skin color (white, brown/mixed, black, or other), educational level (illiterate, incomplete or complete elementary school, incomplete or complete high school, incomplete or complete university education) and marital status (single or without partner, dating, married or with partner, separated, or widowed). Socioeconomic class was analyzed using the Brazilian Criteria of Economic Classification, a system for the Brazilian public that divides the market exclusively in terms of economic class based on the ownership of assets and the education level of the householder^21^. For analysis, social classes were grouped into three categories: A-B, C, and D-E. This social class structure reflects a monthly household income of R$768 (US$ 202) for classes D-E, R$2165 (US$ 569) for class C, and R$11,664 (US$ 3069) for classes A-B^21^.

Participants were asked about their use of contraceptive methods, lifetime condom use and condom use in the first and last sexual intercourse, as well as alcohol and drug use and smoking. They were also asked about sex after drug use, condom use after alcohol and drug use, presence of sexually transmitted infections throughout life (individuals who reported having STIs or who had positive rapid test results for HIV or syphilis at the time of the interview were considered positive), age at first intercourse, number of sexual partners in the last year, same-sex relationships (opposite sex, both, or same sex in the last five years) and types of sexual practices (exclusively vaginal intercourse; vaginal and/or oral intercourse—except anal; vaginal and/or anal intercourse—except oral; vaginal, oral, and anal intercourse). For the sexual practices variable, we asked participants, “When you have sex, what happens?” and questioned them about different practices during sex.

Genital HPV infection was classified as multiple infection (more than one HPV type), high-risk HPV (16, 18, 31, 33, 35, 39, 45, 51, 52, 56, 58, 59, and 68—based on IARC’s classification of oncogenic HPV types^22^), and positive for quadrivalent HPV vaccine types (6, 11, 16, and 18).

### Procedures for biological material collection

Genital biological samples were collected to evaluate the prevalence of different HPV types. For women, cervical samples were collected by trained professionals using a Qiagen HC2 DNA collection device according to the manufacturer’s instructions. Men were instructed to self-collect samples from the entire surface of the scrotum, glans penis/coronal sulcus, and penile shaft using a saline-wetted Dacron swab, under the orientation and supervision of a trained health professional. The swabs were placed in a Digene Specimen Transport Medium.

Oral samples were obtained through mouthwash and gargle cycles, using 10 mL of a standardized commercial mouthwash. The samples were collected from 3 cycles of 5 s each into a Falcon tube identified with a five-digit number bar code and the date of collection^16^. All samples were stored at controlled room temperature and shipped to a central laboratory weekly. More details about the study protocol were previously presented^23^. DNA was extracted from 0.5 mL of specimen transport medium (STM) using magnetic beads for isolation and purification on a robotic system (MagNA Pure LC 2.0; Roche), according to the manufacturer’s extraction instructions^23^. The DNA concentration in the extract was determined using the NanoDrop 2000 (Thermo Scientific™). HPV genotyping was performed using the Roche Linear Array® (LA) genotyping test, which amplifies a 450 bp fragment in the L1 gene^23^. Per reaction, 25 µl working master mix was combined with DNA extract (between 100 and 150 ng) diluted in 25 µl of ultrapure DNase/RNase-Free water^23^. Polymerase chain reaction cycling conditions and hybridization were performed as recommended by the manufacturer. Probes could detect 37 types of HPV simultaneously. The assay incorporates β-globin as an internal control for sample amplification^23^.

### Statistical analysis

The sample size was defined to detect an HPV prevalence of at least 30% with an 80% power. It was calculated according to sex and took into account the study design. Sample size was purposely equal in all regions to maximize diversity in less populated areas^19^.

A descriptive analysis was performed using means and confidence intervals for continuous variables and absolute frequencies for categorical data. The differences between the means were assessed with the t-test, and the chi-squared test was used to evaluate the differences between categorical variables.

The prevalence of oral and genital HPV infection; infection with more than one type of HPV; high-risk HPV; HPV from quadrivalent vaccine; and HPV 6, 11, 16, and 18 separately were assessed according to the type of sex practiced, stratified by biological sex (male and female), and described with their respective 95% confidence intervals (95% CI).

Poisson regression with robust variance analysis was conducted to examine factors associated with HPV infection, adjusting for confounders. For the multivariate analyses, a theoretical framework was structured discriminating hierarchical blocks: type of sex practices (Model 1), gender and age (Model 2), number of sex partners in the last year (Model 3), and same-sex relationships (Model 4). The hierarchical model is an available alternative in epidemiological studies with a large number of covariates^24^.

To adjust the distribution of the sample to the study population, we used a weight adjustment for population size for each capital and by sex. Therefore, all results are reported as weighted. Analyses were performed by using SAS software (Statistical Analysis System, SAS Institute Inc., Cary, NC), version 9.4, and statistical significance was defined as *p* < 0.05.

### Ethical approval

All procedures performed in studies involving human participants were in accordance with the ethical standards of the Moinhos de Vento Hospital research board (Approval No. 1607032) and with the 1964 Helsinki declaration and its later amendments or comparable ethical standards.

## Results

Overall, 6,388 individuals were included (63.6% women). About 27.1% reported having exclusively vaginal intercourse, 49.7% reported having vaginal and/or oral intercourse, 2.8% reported having vaginal and/or anal intercourse, and 20.4% reported having all types of sex. Most participants were brown/mixed color (56.9%), followed by white (23.2%). The mean age of first sexual intercourse was 15.3 years (95% CI 15.2–15.4) and most participants were dating (39.8%) or married/living with a partner (36.5%).

Men reported practicing more of all types of sex (24.7% vs. 18.2%, *p* = 0.0032) than women (Table 1). Participants with higher education and socioeconomic class, as well as those who were white, reported more oral sex. As for behavioral characteristics (Table 2), smokers practiced more anal sex and all types of sex than former smokers or those who never smoked. Condom use at first intercourse and current condom use were not significantly different between sexual practices (data not shown). However, participants who reported using condoms during their last sexual intercourse had more exclusively vaginal sex. Individuals with a higher number of partners in the last year also practiced more of all types of sex and oral sex. Participants who reported sexual intercourse with a partner of the same sex practiced more anal sex (14.9% vs. 2.3%, *p* < 0.0001) and all types of sex (32.7% vs. 18.6%, *p* = 0.0061).

**Table 1:** Distribution of sociodemographic characteristics of the population studied, according to types of sex practices (n = 6388).

	n (%)							
	Exclusive vaginal sex	*p *value	Oral sex	*p *value	Anal sex	p-value	Oral, vaginal, and anal sex	*p *value
**Gender**								
Male (1120)	207 (22.4)	0.059	480 (49.8)	0.9662	22 (3.1)	0.6638	277 (24.7)	0.0032
Female (5268)	1460 (29.6)		2486 (49.6)		127 (2.6)		931 (18.2)	
**Age (years)**								
16–21 (3422)	958 (29.4)	0.0133	1565 (49.1)	0.5753	82 (2.7)	0.8579	597 (18.8)	0.0690
22–25 (2966)	709 (24.5)		1401 (50.4)		67 (2.9)		611 (22.2)	
**Skin color**								
White (1519)	309 (20.9)	< 0.0001	817 (58.2)	< 0.0001	24 (1.5)	0.0755	283 (19.4)	0.9449
Brown/mixed (3671)	998 (26.9)		1608 (49.0)		103 (3.5)		193 (20.6)	
Black (1013)	286 (32.4)		468 (44.0)		18 (2.7)		704 (20.9)	
Other (151)	60 (48.5)		64 (30.7)		4 (1.2)		20 (19.6)	
**Relationship status**								
Single /without partner (1339)	325 (24.0)	< 0.0001	622 (53.5)	< 0.0001	25 (2.5)	0.9102	225 (20.0)	0.5624
Dating (2403)	500 (22.7)		1309 (55.1)		50 (3.0)		422 (19.2)	
Married /with partner (2570)	821 (33.7)		1006 (41.5)		72 (2.8)		545 (22.0)	
Separated/widowed (75)	20 (26.9)		29 (51.2)		2 (1.1)		16 (20.8)	
**Socioeconomic status**								
A-B (1088)	162 (15.8)	< 0.0001	632 (60.8)	< 0.0001	13 (1.4)	0.1810	213 (22.0)	0.7394
C (3402)	873 (25.2)		1656 (51.8)		74 (3.1)		619 (19.9)	
D-E (1898)	632 (37.4)		678 (39.0)		62 (3.1)		376 (20.5)	
**Educational level**								
Illiterate/elementary (1373)	510 (39.9)	< 0.0001	450 (36.3)	< 0.0001	44 (2.9)	0.0029	285 (20.9)	0.1065
Secondary school (3571)	949 (26.2)		1649 (51.4)		91 (3.5)		658 (18.9)	
Higher education (1443)	207 (14.4)		867 (60.8)		14 (0.7)		265 (24.1)

**Table 2:** Distribution of behavioral characteristics of the studied population, according to types of sex practices (n = 6388).

	n (%) or mean (95% CI)							
	Vaginal sex	*p *value	Oral sex	*p *value	Anal sex	*p *value	Oral, vaginal, and anal sex	*p *value
**Smoking**								
Never smoked (4389)	1215 (28.2)	0.1222	2036 (51.4)	0.0868	100 (2.2)	< 0.0001	754 (18.2)	0.0041
Current smoker (807)	168 (22.1)		357 (44.7)		20 (7.0)		215 (26.2)	
Former smoker (1192)	284 (27.0)		573 (47.2)		29 (1.9)		239 (23.9)	
**Alcohol consumption during life**								
No (1890)	660 (38.6)	< 0.0001	723 (44.6)	0.0051	52 (2.8)	0.9786	13 (14.0)	< .0001
Yes (4492)	1006 (22.4)		2241 (51.8)		97 (2.8)		84 (23.0)	
**Drug use during life**								
No (4781)	1382 (30.7)	< 0.0001	2138 (48.5)	0.1167	121 (2.5)	0.3483	51 (18.3)	0.0006
Yes (1601)	284 (18.1)		826 (52.7)		28 (3.5)		46 (25.7)	
**Sex practice after drug or alcohol use**								
No (5129)	1498 (30.9)	< 0.0001	2311 (48.2)	0.0022	126 (3.0)	0.0780	860 (17.9)	< .0001
Yes (1148)	146 (11.4)		604 (57.3)		20 (1.7)		325 (29.6)	
**Condom use under the influence of alcohol or drugs**								
Never (386)	60 (14.6)	0.2244	201 (55.8)	0.8437	8 (1.5)	0.0326	109 (28.1)	0.7200
Sometimes (519)	54 (9.2)		261 (56.9)		12 (2.3)		165 (31.6)	
Always (255)	37 (12.8)		143 (60.1)		2 (0.1)		55 (27.0)	
**Condom use in the last intercourse**								
No (3954)	958 (25.2)	0.0119	1868 (50.1)	0.6209	92 (2.6)	0.5907	832 (22.0)	0.0215
Yes (2409)	704 (30.5)		1093 (48.9)		57 (3.1)		372 (17.5)	
**Number of sex partners in the last year**								
< 2 (4509)	1362 (32.6)	< 0.0001	2047 (48.1)	0.0260	113 (2.6)	0.4643	754 (16.7)	< 0.0001
≥ 2 (1636)	257 (14.2)		841 (54.3)		33 (3.3)		397 (28.2)	
**Same-sex relationships**								
Opposite sex (5336)	1481 (28.6)	< 0.0001	2551 (50.5)	0.4782	124 (2.3)	< 0.0001	71 (18.6)	0.0061
Both or same sex (282)	21 (6.4)		131 (46.1)		5 (14.9)		7 (32.7)	
**Presence of self-reported STI or positive rapid test for HIV or syphilis**								
No (5341)	1477 (28.6)	0.0031	2489 (49.7)	0.8831	123 (2.6)	0.5189	969 (19.1)	0.0047
Yes (702)	137 (19.5)		328 (49.1)		14 (3.6)		166 (27.7)	

In total, 27.41% of 4,761 unvaccinated women reported having anal sex. No significant differences between those who practiced or did not practice anal sex were found between genital HPV (56.8% vs. 52.73%, *p* = 0.114) and high-risk HPV prevalence (35.7% vs. 35.22, *p* = 0.848) (data not shown).

The prevalence of any type of cervical HPV and 4vHPV types was significantly higher for women who practiced oral sex and all types of sex. HR-HPV was higher in women than men who practiced oral (41.12% vs. 31.31%, *p* = 0.0133) or vaginal sex (35.23% vs. 20.58%, *p* = 0.0037) but was not significantly different between types of sex among each biological sex (Table 3). Still, men who practiced anal sex had higher prevalence of genital 4vHPV types than women (31.86% vs. 8.91%, *p* = 0.0397). Among men, overall HPV (65.87%) and multiple infection (39.34%) prevalence were significantly higher in those who practiced all types of sex.

**Table 3:** Prevalence of genital, high-risk, multiple HPV infections, and vaccine types with 95% confidence interval (CI), according to types of sex practiced and by gender.

	Any HPV type	HR-HPV	Multiple infection	Quadrivalent vaccine
	% (95%CI)	% (95%CI)	% (95%CI)	% (95%CI)
**Female**				
Exclusive vaginal sex	50.85 (47.01–54.68)	35.23 (31.54–38.92)^b^	28.86 (25.43–32.30)	13.71 (11.10–16.31)
Oral sex	57.85 (54.88–60.82)^a^	41.12 (38.14–44.10)^c^	36.87 (33.93–39.80)^d^	18.31 (15.96–20.67)^e^
Anal sex	48.84 (36.11–61.57)	41.68 (29.05–54.30)	30.19 (18.14–42.24)	8.91 (2.91–14.92)^f^
Oral, vaginal, and anal sex	53.63 (48.91–58.34)	37.38 (32.88–41.88)	27.97 (23.89–32.06)	14.20 (11.06–17.33)
	*p = 0.0238	*p = 0.0834	*p = 0.0005	*p = 0.0079
**Male**				
Exclusive vaginal sex	46.78 (35.97–57.59)	20.58 (12.58–28.58)^b^	20.78 (12.53–29.03)	10.75 (4.79–16.70)
Oral sex	48.67 (41.21–56.13)^a^	31.31 (24.48–38.14)^c^	25.17 (19.17–31.17)^d^	11.57 (7.69–15.45)^e^
Anal sex	47.94 (15.36–80.52)	26.05 (0.00–56.21)	34.39 (2.97–65.81)	31.86 (0.87–62.85)^f^
Oral, vaginal, and anal sex	65.87 (56.48–75.26)	37.79 (27.35–48.24)	39.34 (28.84–49.85)	15.97 (8.89–23.04)
	*p = 0.0411	*p = 0.1266	*p = 0.0375	*p = 0.1589

*P value indicates whether there is a difference in the prevalence of HPV between the different types of sex practiced, in each stratum (for men and women).

^a,b,c,d,e,f^Indicates that there is a significant difference in the prevalence of HPV between men and women for each type of sex practiced.

When adjusted for confounding factors, type of sex practiced was not significantly associated with HPV infection (Table 4). Women had higher rates of HPV than men, and being 22 years of age or older was a protective factor in all multivariate models. HPV infection was 1.41 times higher in those who had two or more partners in the last year [PR 1.41 (CI95% 1.41, 1.28–1.55)].

**Table 4:** Multivariate analyses of factors associated with overall HPV infection.

	Model 1	Model 2	Model 3	Model 4
	Prevalence ratio (95% confidence interval)			
**Type of sex**				
Exclusive vaginal sex	1	1	1	1
Oral sex	1.10 (0.99–1.22)	1.11 (1.00–1.23)	1.03 (0.93–1.14)	1.02 (0.92–1.13)
Anal sex	0.97 (0.71–1.34)	0.98 (0.72- 1.36)	0.92 (0.65–1.31)	0.92 (0.65–1.31)
Oral, vaginal, and anal sex	1.18 (1.05–1.33)	1.20 (1.07–1.35)	1.09 (0.97–1.23)	1.09 (0.96–1.23)
**Gender**				
Male		1	1	1
Female		1.06 (0.96–1.18)	1.17 (1.04–1.31)	1.17 (1.04–1.33)
**Age (years)**				
16–21		1	1	1
22–25		0.89 (0.81–0.96)	0.90 (0.83–0.98)	0.91 (0.84–0.99)
**Number of sex partners in the last year**				
< 2				1
≥ 2			1.36 (1.24–1.49)	1.41 (1.28–1.55)
**Same-sex relationships**				
Opposite sex				1
Both or same sex				0.96 (0.77–1.19)

Poisson regression with robust variance.

**Figure 1:**
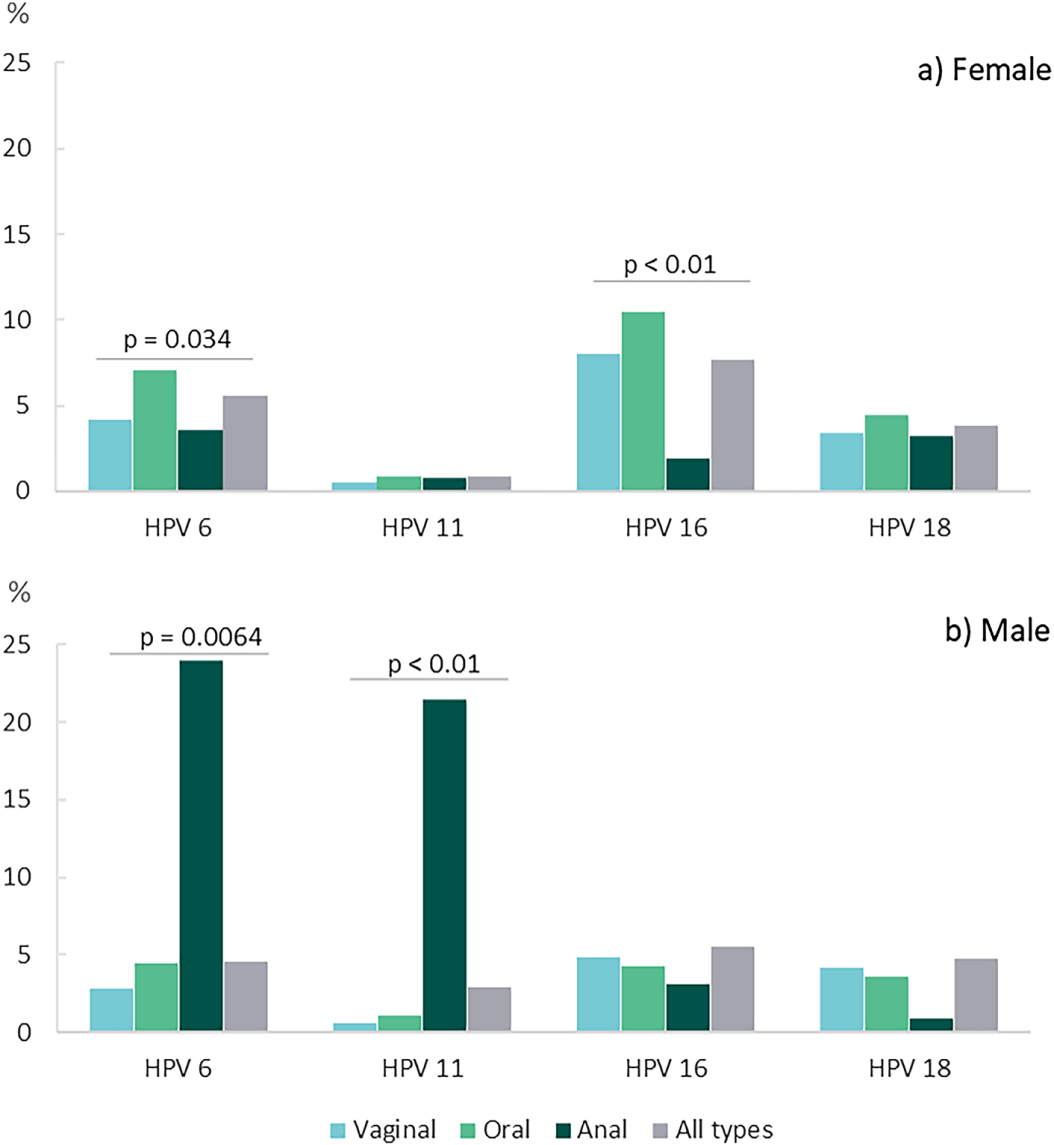
Prevalence of genital HPV types according to sexual practices in female and male types of sex practices (n = 6388).

When we look at the isolated HPV types, women who practiced oral sex had higher prevalence of HPV 6 (7.1%) and 16 (10.5%) than those who practiced vaginal (4.1% and 8.0%), anal (3.5% and 1.9%), or all types of sex. On the other hand, men who practiced anal sex had significantly higher prevalence of HPV 6 (23.96%) and 11 (21.49%) (Fig. 1).

Among oral samples from 4313 participants, only 0.06% and 0.10% were positive for HPV 16 and HPV 18, respectively. There was no significant difference in the prevalence of oral HPV by type of sex (*p* = 0.917): 2.08% among those who practiced exclusively vaginal sex, 2.04% for those who practiced oral sex, 0.64% for those who practiced anal sex, and 2.28% all types of sex. Furthermore, 47 individuals (1.36%, 95% CI 0.65–2.07) had both oral and genital HPV infection (data not shown).

## Discussion

This study helps to fill a gap in the literature, providing information on the prevalence of genital and oral HPV according to the sexual practices reported by sexually active young people in Brazil. Among the main results, we can highlight that age and number of sexual partners in the last year were more important determinants of HPV infection than type of sex practiced. Oral sex was associated with white skin color, marital status dating, higher socioeconomic status, and higher educational level. HPV 6 and 16 were associated with exclusively vaginal sex among women, and types 6 and 11 were associated with anal sex among men. On the other hand, the prevalence of oral HPV was low and not associated with sexual practices.

A significant difference in genital HPV infection according to sexual practices was found when the analysis was stratified by biological sex. Women had significantly higher prevalence in all the analyzed outcomes (overall HPV, multiple infection, HR-HPV, and HPV vaccine types) than men. This may be because the susceptibility to infection varies with the epithelium. HPV infections are believed to occur following wounding of epithelium or mucosa and subsequent access of virus to basal epithelial cells. Natural transmission of cutaneous infections likely involves physical contact, allowing virus to be shed into the wounded site, or occurs following mechanical wounding during sexual intercourse for vaginal and anal infections^25^.

As in previous studies^26,27^, no association was found between oral HPV and any particular sexual practice. The low prevalence may be one of the reasons for the lack of association. Even so, it is noteworthy that the number of lifetime sexual partners may be more relevant than the type of sexual practice^28^. Gester et al. evaluated unprotected sex and passive oral sex, and the prevalence of oral HPV was no longer apparent after adjustments^29^.

Besides a significant difference in transmission between the sexes, some studies also suggest selective transmission of different HPV types. HPV 16 could be the most readily transmitted type, not because of its intrinsic transmissibility^7^, but instead due to pathogenic differences. The HITCH cohort study, which enrolled young women (18–24 years old) and their male partners of over 4 months, indicated that HPV16/18 did not have particularly high transmission rates relative to other HPV types^30^. In the present study, only genital HPV 11 was significantly more common in those who practiced anal sex, which is interesting since this HPV type can cause anogenital lesions, and the risk for anal HPV infection in individuals with previous genital HPV seems to be higher than in individuals without an infection^31^.

This study confirmed findings from previous studies’ that showed the practice of anal sex was associated with certain behaviors, such as current smoking and having sex with partners of the same sex^32^, while oral sex was more common among those who had more partners in the last year and those who had a history of consuming alcohol and drugs. Contrarily, heterosexual couples who reported always using condoms had lower HPV transmission rates^30^. Additionally, condom use in the last intercourse was significantly more frequent in those who reported exclusively vaginal sex, which may explain the lower prevalence of HPV in this group.

It is known that vaccination of sexually active women reduces transmission of different HPV types in heterosexual couples^33^. On the other hand, receptive anal sex is strongly associated with HPV detection in the anal canal in men who have sex with men (MSM)^34^. Nyitray et al. (2016) showed that persistence of high-risk anal HPV is associated with the number of male anal sex partners and inversely associated with the number of female sex partners^34^. The impact of vaccination in men goes beyond protecting the female population and directly benefits men themselves, as they showed a high prevalence of HPV vaccine types in the present study^35^. Since nearly 90% of anal cancers have been estimated to be caused by HPV^36^ and HPV 16 is the most carcinogenic type in the anus, vaccination could help prevent anal cancer in this population^37^. A systematic review and meta-analysis found that overall prevalence of anal, penile, and oral HPV infection among men who have sex with men were 78.4%, 36.2%, and 15.4%, respectively, with higher rates in HIV-positive than HIV-negative MSM. The most frequent HPV high-risk type detected in the anus, penis, and oral cavity was HPV 16 (19.9%, 4.9% and 3.1%, respectively)^38^.

Knowledge about the roles of different sexual practices in HPV infection as well as the most prevalent HPV types is important to inform policy not only in Brazil but globally. Since the HPV vaccine and HPV testing are becoming widely implemented in many countries for cervical cancer screening^39^, the awareness of HPV-associated cancers has increased. Nevertheless, many people might have doubts and anxieties regarding HPV transmission^40^. Clinicians and public health workers should be able to inform the public on the modes of transmission and prevention strategies.

Despite the importance of this study, some limitations should be noted. The types of sexual practices were always categorized including vaginal sex, because we did not have enough data evaluating exclusive oral and anal intercourse. The practice of oral sex may have been reported as either “doing” or “receiving.” It is worth mentioning that the prevalence of anal HPV was not analyzed. In addition, a cross-sectional study could reflect transmission in previous or concurrent relationships. Further studies with larger sample sizes and with both sexual partners are needed to investigate the transmission modes for specific HPV types.

Given the high prevalence of HPV in Brazilian young adults, it is important to assess how the virus can be transmitted. Few studies have assessed the presence of HPV infection at different anatomic sites simultaneously. The present study tries to elucidate this topic by assessing HPV prevalence according to sexual practice. The type of sex practiced was not significantly associated with cervical or oral HPV infection. While this study offers new information on the prevalence of genital and oral HPV infection, other analyses should be developed assessing the relationship between HPV infection in different sites of body and sexual practices in the population.

## Data Availability

The datasets analyzed during the current study are available from the corresponding author on reasonable request.
