# Author Correction: Sexual practices and HPV infection in unvaccinated young adults

**DOI:** 10.1038/s41598-022-18818-0

**Published:** 2022-08-25

**Authors:** Sílvia Pauli, Natália Luiza Kops, Marina Bessel, Luisa Lina Villa, Flávia Moreno Alves Souza, Gerson Fernando Mendes Pereira, Fernando Neves Hugo, POP-Brazil Study Group, Juliana Comerlato, Isabel Bandeira, Bruna Fernandes, Tiago Fetzner, Milena Mantelli Dall Soto, Thais Baptista, Barbara Pereira Mello, Giana Mota, Eliana Wendland

**Affiliations:** 1 Hospital Moinhos de Vento, Rua Ramiro Barcelos 910, Porto Alegre, RS 90035-004 Brazil; 2 Department of Radiology and Oncology, Medical School, Universidade de São Paulo (USP), and Instituto do Câncer do Estado de São Paulo (ICESP), São Paulo, SP Brazil; 3 Department of Chronic Conditions and Sexually Transmitted Infections, Ministry of Health, Brasília, DF Brazil; 4 Department of Preventive and Social Dentistry, Universidade Federal do Rio Grande do Sul, Porto Alegre, RS Brazil; 5 Department of Public Health, Universidade Federal de Ciências da Saúde de Porto Alegre (UFCSPA), Porto Alegre, RS Brazil

Correction to: *Scientific Reports* 10.1038/s41598-022-15088-8, published online 20 July 2022

The original version of this Article contained an error in the spelling of the author Eliana Wendland which was incorrectly given as Eliana Marcia Wendland.

The original Article has been corrected.

